# A study of interactive robot architecture through the practical implementation of conversational android

**DOI:** 10.3389/frobt.2022.905030

**Published:** 2022-10-11

**Authors:** Takashi Minato, Kurima Sakai, Takahisa Uchida, Hiroshi Ishiguro

**Affiliations:** ^1^ Guardian Robot Project, RIKEN, Kyoto, Japan; ^2^ Hiroshi Ishiguro Laboratories, Advanced Telecommunications Research Institute International, Kyoto, Japan; ^3^ Guraduate School of Engineering Science, Osaka University, Osaka, Japan

**Keywords:** conversational robot, android, daily dialogue, multimodal Turing test, architecture

## Abstract

This study shows an autonomous android robot that can have a natural daily dialogue with humans. The dialogue system for daily dialogue is different from a task-oriented dialogue system in that it is not given a clear purpose or the necessary information. That is, it needs to generate an utterance in a situation where there is no clear request from humans. Therefore, to continue a dialogue with a consistent content, it is necessary to essentially change the design policy of dialogue management compared with the existing dialogue system. The purpose of our study is to constructively find out the dialogue system architecture for realizing daily dialogue through implementing an autonomous dialogue robot capable of daily natural dialogue. We defined the android’s desire necessary for daily dialogue and the dialogue management system in which the android changes its internal (mental) states in accordance to the desire and partner’s behavior and chooses a dialogue topic suitable for the current situation. The developed android could continue daily dialogue for about 10 min in the scene where the robot and partner met for the first time in the experiment. Moreover, a multimodal Turing test has shown that half of the participants had felt that the android was remotely controlled to some degree, that is, the android’s behavior was humanlike. This result suggests that the system construction method assumed in this study is an effective approach to realize daily dialogue, and the study discusses the system architecture for daily dialogue.

## 1 Introduction

Until now, the dialogue (verbal communication) function of smart speakers (e.g., Amazon Alexa and Google Nest), robots (e.g., Bazzano and Lamberti, 2018; Aceta et al., 2022), and computer graphics (CG) agents (e.g., Nishimura, et al., 2003; Uchida and Onogi, 2013) has been only a protocol for exchanging information with human users. In a society where robots coexist with humans and robots become humans’ companions, humans and robots will have daily dialogues. Unlike dialogues aimed at providing or exchanging information (task-oriented dialogue), daily dialogue additionally involves mutual understanding and empathy (Okada et al., 1998) with unclear purposes (i.e., non–task-oriented contents). A dialogue system for the purpose of information transmission requires a technology that correctly recognizes user’s requests and responds appropriately. Recently, these systems have been developed through data-driven technologies such as deep learning. The purpose of these systems is basically given by users and designers who design services using the interactive systems, and these systems are required to respond to the user’s any requests related to the services. However, in daily dialogue, the dialogue system is not given a clear purpose or the necessary information. That is, the daily dialogue system needs to generate an utterance in a situation where there is no clear request from the user. Therefore, it is necessary to design a system to proactively interact with the user. In other words, the system needs to independently behave in the dialogue. In another aspect, the task-oriented dialogue basically aims at providing objective information. On the other hand, speakers exchange not only objective information but also their subjective information in the daily dialogue. This suggests that the daily dialogue system needs to have its own values to generate subjective information, that is, to behave independently from the users.

The existing non–task-oriented dialogue systems such as chat systems have generated appropriate responses to users’ utterances based on a huge dialogue database (Shibata et al., 2009; Bessho et al., 2012; Higashinaka et al., 2016), so there was nothing to drive such independent utterances. As a result, the context easily breaks down [even the latest chatbots (Adiwardana et al., 2020) can only be continued for 10–30 turns in a consistent context]. Therefore, in order to realize the context with a consistent and continuous content in a daily dialogue, it is necessary to essentially change the design policy of dialogue management compared with the existing dialogue system.

So far, our research group has developed a system that enables daily dialogue using an android robot that closely resembles a human. The purpose of this study is to constructively find out the dialogue system architecture for realizing daily dialogue. Therefore, we implemented an autonomous dialogue robot capable of daily humanlike dialogue (though it works in a limited situation) and investigated the developed architecture.

The significance of this study is as follows:

1) This study has implemented various interactions that occur in daily dialogue with various modalities, although in a limited situation (first-meeting dialogue).
2) This study constitutively confirms the dialogue system architecture for realizing daily dialogue, which is different from the existing dialogue systems.

## 2 Functions needed for daily dialogue

### 2.1 Desire-based topic management

Task-oriented dialogues such as voice guidance (Adachi et al., 2003) and discussion (Sakai et al., 2020) have clear goals, and the dialogue strategy is designed to achieve the goal. In these dialogues, the conveyed information through the dialogue is important, and the existing studies focus on the design of the dialogue strategy to obtain and provide information necessary to achieve the goal. On the other hand, the content of the daily dialogue such as a chat does not have a clear purpose. The important function of the daily dialogue is not only information transmission but also the empathizing with each other and is one means of communication for gaining understanding and empathy with others (Okada et al., 1998). Therefore, through not only letting the robot talk to meet the humans’ requests but also giving it the same desires that the humans express in dialogues, it will become possible for the robot to have a natural dialogue for gaining understanding and empathize with others.

During discourse analysis, we found a study investigating the desires that appear in daily human dialogues in domestic situations (Haruki, 2013). In this study, it is considered that daily dialogue is governed by the desires for knowledge acquisition, empathy, pleasant orientation, discomfort avoidance, approval, communication engagement, and communication maintenance, that is, daily dialogue is controlled by human desire. It is also considered that those desires shape Grice’s conversational maxims (Grice, 1989). Regarding task-oriented dialogues, it can be said that the desires for knowledge acquisition and communication maintenance drive the dialogues. The other desires are related to gaining understanding and empathy with others which are important functions of daily dialogue.

The daily dialogue is non–task oriented, but it is not without patterns in context development. For example, it has been shown that a certain routine can be seen in the development of topics such as the first-meeting dialogue (Kellerman and Lim, 1989). In addition, it has been shown that topics are derived and developed in a hierarchical structure (Murakami and Kumatoridani, 1995), and there is a pattern in topic development (Kawachi, 2009) in daily dialogues. Therefore, the basic structure of daily dialogues include utterance generation and action generation based on the desires appearing in human daily dialogues, where the utterance content is constrained by the topic, and the topic develops according to a certain pattern.

### 2.2 Utterance generation based on dialogue topic

Each daily dialogue is composed of task-oriented interactions. As aforementioned, the utterances are generated while maintaining the context within the weak constraint of the topic. In task-oriented dialogue systems, the constraints of the topic are often not defined, therefore the context could break down in a few turns. The daily dialogue system requires an utterance generation mechanism based on topical constraints. In addition, it is known that there is a fixed speech pattern for a dialogue according to the topic. Studies examining daily dialogues have shown that there is a fixed speech chain pattern (utterance sequence organizations) (Tsutsui, 2012). The existing task-oriented dialogue systems generate responses to human requests, but in a daily dialogue, the ability for the system to proactively start talking according to its desire is also required. For that purpose, it is necessary to describe a dialogue scenario of a certain length in the topic and design the dialogue system architecture to generate utterances according to the scenario.

This topic-based utterance generation is helpful for the robustness of the dialogue system. It has been shown that the domain knowledge modeled with dialogue topics is important for maintaining robustness of the system and improving recognition accuracy of spoken utterances (Jokinen et al., 1998).

### 2.3 Subjective preference and experience of a robot

In task-oriented dialogues, the objective information exchange is the main purpose for task achievement, but in daily dialogues, an exchange of subjective information rather than objective information mainly occurs. It has been shown that the subjective opinion exchange is the main activity, especially in situations where the dialogue is lively (Tokuhisa and Terashima, 2006). In order to realize such a dialogue, it is necessary to give the dialogue system subjective experiences and preferences and design a mechanism for utterance generation based on these.

### 2.4 Turn-taking management

In the dialogue with an agent who responds to questions, it is desirable that the user who asks the question takes the initiative in the turn. That is, the agent takes a turn after the user’s question is finished, and if the user’s question barges in during the agent’s utterance, the agent should stop speaking and listen to the user’s utterance. However, in daily dialogue, it is not always possible to clearly determine when the dialogue participants take turns, as in the question–answering protocol. Speech overlap may occur in situations where it is unclear who should take the turn. At the time of silence or utterance overlap, who takes the turn is adjusted according to the situation (Ikoma, 1996).

In addition, in daily dialogue, a dialogue breakdown may occur due to misunderstanding of the other’s utterance or misunderstanding of meaning (Komatani and Kawahara, 2000). In a dialogue system for the purpose of providing information, when such a breakdown occurs, it is often the case where the user is simply prompted to speak again. However, in daily dialogue, recovery from a dialogue breakdown depends on the situation in which the breakdown occurs and on the emotions of the interlocutor. If the dialogue system makes a statement that encourages a similar recurrence every time it fails, it is considered that the dialogue is unnatural and the user’s willingness to interact is diminished. Even in the case of turn adjustment and recovery from the breakdown, it is necessary to have a mechanism that speaks according to the situation and the internal state of the robot.

On the other hand, interrupting utterances from the system side may be required, that is, nodding and backchannel utterances. In daily dialogue, while listening to the other person’s story, we can see backchannels and behaviors that indicate conformity, empathy, and nonconformity (Gratch et al., 2006; Levitan and Hirschberg, 2011; Kawahara et al., 2016). This has the effect of increasing the satisfaction of the speaker and the willingness to continue the dialogue. Even in a dialogue system, a mechanism for giving backchannel utterances during the user’s utterance is indispensable for realizing a natural dialogue.

### 2.5 Mutual understanding and empathy

An important interaction in daily dialogue is to exchange subjective impressions and opinions about experiences (Hirano et al., 2016) and exchange preferences and values with each other (Hirata, 2012). In general, for understanding the dialogue partner, the perspective taking is necessary as the Theory of Mind (Premack and Woodruff, 1978) suggests. [Some studies have developed autonomous agents to have cooperative tasks with humans designed according to the Theory of Mind (Minoya et al., 2011; Buehler and Weisswange, 2020).] Empathy is also necessary since neuroscience has shown that there are two pathways on how the human brain processes understanding others: an affective route for the direct sharing of others’ emotions (empathy) (de Vignemont and Singer, 2006) and a cognitive route for representing and reasoning about others’ states (Theory of Mind). In addition, Grice’s conversational maxims (Grice, 1989) and the relevance theory (Sperber and Wilson 1995) provide linguistic clues for understanding others. Even if daily dialogue is implemented by a scenario-based dialogue system, it is necessary to understand the minimum subjective opinions, preferences, and values in user utterances and empathize with the user. As a language comprehension ability, the method of extracting a specific keyword in the utterance can be easily implemented, but it is difficult to realize the aforementioned interaction. First, the response required for exchanging values is expressing agreement or disagreement (Ihara and Kobayashi, 2005), and for that purpose, it is necessary to understand at least the sentiment of whether the user’s utterance is positive or negative. In exchange of experiences, the dialogue system needs to understand the experience and subjective opinions, as well as express agreeable or disagreeable responses to it. Although information about experiences is complicated, the information exchanged in a dialogue is mainly 1H5W (what, why, when, how, where, and who) information, and the experience description and recognition capabilities by the 1H5W frame description are all that is minimally required (Kim et al., 2010). Subjective impressions and opinions about a user’s experience can then be recognized by describing the user’s experience utterances through such a semantic network. In the exchange of preferences, an abstract understanding of preferences allows us to talk about what we like and whether it resembles our own preferences. For this purpose, it is necessary to build a model of the user’s preference from the user’s utterances and incorporate it into a system that can estimate the preferences based on it. To show empathy with the user, it is necessary to recognize or estimate the user’s emotion and to generate an emotion generation model for the robot (e.g., Becker and Wachsmuth, 2010).

### 2.6 Relationship building

In daily dialogue, relationships with the other party are built through alignment of each other’s values (Dimulescu, 2017). The purpose of social interaction is to maintain and strengthen relationships with others (Malinowski 1994; Dunbar, 1996; Eggins and Slade, 1997). Therefore, the necessary function is to memorize the other’s information and generate utterances based on it, estimate the other’s preferences and interests, and estimate the other’s emotions in order to empathize with the other. In addition, the robot has to express emotions through voice and movement and to show empathy.

### 2.7 Emotional expression

The study of Haruki (2013) describes the desires for knowledge acquisition, empathy, and pleasant orientation those appear in daily dialogues. These desires inevitably involve emotional expressions. The study that analyzed emotions in a first-encounter dialogue suggests that some emotions are related to specific communicative functions, such as giving feedback and managing turns (Navarretta, 2012). Moreover, it has been shown that affective and cooperative utterances are significant in creating enthusiastic non–task-oriented dialogues, which are necessary for maintaining daily dialogues (Tokuhisa and Terashima, 2006).

## 3 Related works

Many dialogue systems have been developed, such as text-based chat systems, multimodal dialogue systems using CG agents, and dialogue robots. Here, this study describes the dialogue systems that relate to the functions necessary for daily dialogues.

### 3.1 Action decision based on intention and desire

A daily dialogue is a mixed initiative, in which both participants take the initiative. In task-oriented dialogues, it is sufficient to build a user-initiative system that provides answers to user’s questions and requests, whereas in daily dialogues, the system needs to speak with initiative. Furthermore, since daily dialogues do not have a specific task, it is necessary to develop a system that proactively behaves according to its own motives rather than reacting to something that changes in the environment. As a desire-based dialogue system, the BDI-based model was developed based on the Bratman’s theory of human practical reasoning (Bratman, 1987). According to the beliefs and desires of the system, the goal to be achieved and its means (plan) are selected from the plan library, and the action is executed according to the intention to accomplish the plan. The rational action can be generated according to the desire and BDI logic.

Zwaan et al. (2012) developed a conversational agent that provides social support for users with cyberbullying problems using the BDI model. The support that the agent should perform during a dialogue is logically inferred using the BDI model. The BDI model is suitable for rational action planning up to the goal, but the agent does not behave proactively with desire in the absence of task constraints. Yorke-Smith et al. (2012) developed a personal assistant capable of voice dialogue. Like the aforementioned system, the agent’s assistance can be inferred using the BDI model and the agent behaves proactively. In this study, the agent’s desires are also used for assistance tasks and are not designed to proactively generate daily interactions. Ushida et al. (1998) developed a mind and consciousness model that autonomously determines behavior using a mental mechanism that uses desires and emotions for value judgments and a consciousness mechanism for selective attention, reflex, and deliberation, and they developed a dialogue agent based on this. This agent has a desire concerning hobbies and preferences (such as wanting the user to like music) and selects utterances by the slot filling method so as to satisfy the desire. But in the implemented system, the desire can be satisfied with one turn interaction, so it is rather a dialogue-like question-answering protocol. Moulin-Frier et al. (2018) proposed a robot cognitive architecture based on the Distributed Adaptive Control theory of mind and brain. The robot was endowed with an intrinsic motivation, which enabled the robot to learn while actively interacting with humans. This architecture generated the robot’s proactive behavior in a non–task-oriented situation, but it was not applied to daily dialogues.

### 3.2 Utterance generation based on dialogue topic

Script-based dialogue management systems are adopted in mainly task-oriented dialogue systems (e.g., Rudnicky and Xu, 1999; Bohus and Rudnicky, 2009), where the scripts can be predesigned according to the task domain knowledge. On the other hand, it is not possible to design scripts covering a wide range of daily dialogue topics. However, to maintain the context of the dialogue, a script-based dialogue system is useful. Matsusaka et al. (2010) studied an extensible script system to improve the scalability of the script-based dialogue system.

### 3.3 Turn-taking management

In many spoken dialogue systems, utterances and actions are regulated by chunks for each turn of the speaker. In other words, it is assumed that the turn-taking will be smooth. In the text dialogue system, the system is designed so that speech overlap does not occur. However, in daily dialogues, utterances and actions are decided and executed in real time, and if there is an utterance of the other party during the speaker’s speech, the dialogue is interrupted and the topic is changed, or a new topic is made before the other party’s speech ends.

Thorisson (1999) developed a dialogue system that combines multiple control layers with a fast cycle that reacts quickly and a slow cycle that controls dialogue content. It successfully realized real-time turn-taking even if the user interrupts the agent's speech even though it dealt with task-oriented dialogue.

### 3.4 Dialogue understanding and mutual understanding

Understanding the meaning of sentences has been studied in the field of natural language understanding (NLU), but unlike task-oriented dialogue systems, it is difficult to understand the meaning and generate utterances in open-domain dialogues such as daily dialogues. Therefore, many systems based on end-to-end learning using a huge database of interpersonal dialogue have been developed (Li et al., 2015; Shang et al., 2015; Sordoni et al., 2015; Vinyals and Le, 2015). A huge dialogue database is effective for generating semantically appropriate utterances; however, it is still difficult to maintain the consistency of the dialogue context. Meena (Adiwardana et al., 2020) has the best performance of any chatbot so far with respect to the evaluation metric called Sensibleness and Specificity Average (SSA), which captures the key elements of a good conversation (sensibleness averagely evaluates whether the meaning of the utterance is logically correct or the meaning of the specificity utterance is context limited). However, it is still difficult to continue the dialogue for a long turn [even the latest chatbots can only be evaluated in 10–30 turns (Adiwardana et al., 2020)].

Narimatsu et al. (2019) developed a chatbot system that focuses on understanding the user’s experience as a minimum requirement for daily dialogue. Focusing on the fact that the user’s experience can be expressed by 1H5W and their impression, the 1H5W information in the user’s utterance is used as the dialogue context. For example, when the user talks about an experience of doing something, the system asks him when and where he experienced it if he does not talk about this. Then, the dialogue about the context of the user’s experience can be continued. In addition, if the system has the same experience and impressions about it, it is possible to empathize with the user’s utterances. The user’s experience can be acquired by 1H5W questions, but if the system repeats asking the user’s experience by 1H5W questions, it becomes annoying for the user. In information-seeking dialogues, optimizing the number of questions has been tried by using an inference system (e.g., Katsumi et al., 2019). The inference-based system is much more necessary for a daily dialogue to maintain a comfortable dialogue.

### 3.5 Relationship building based on subjective utterance

Kasap and Magnenat-Thalmann (2012) developed a dialogue system that has an episodic memory about sharing experience with users, and a tutoring agent that interacts with users based on this. Although it is not a daily dialogue, it has been shown that there is an effect in enhancing the user’s engagement by referring to the shared experience with the user. Kobayashi and Hagiwara (2016) developed a non–task-oriented dialogue system that considers users’ preferences and their relationships. Through dialogue with multiple people, it collects information on people’s preferences and proactively refers to other people who have the same preferences with respect to the user’s preferences. It has been clarified that the liking and satisfaction with the dialogue system is increased by referring to the information of others.

### 3.6 Emotion recognition and expression

There are many studies on the expression of emotions of robots such as Kismet (Breazeal, 2003). They built an internal state model for emotion generation and expressed emotions by facial expressions and postures. The emotional expressions contribute to enhancing the engagement of user interaction and forming a rapport. Moreover, it has been shown that the empathic behaviors of the agent contribute to alleviate the user’s mental stress (Prendingner et al., 2005). SimSensei Kiosk (DeVault et al., 2014) recognizes facial expressions from the user’s face image and estimates the positive or negative valence of the user’s utterance and produces a virtual human interviewer that can express humanlike gestures and facial expressions although the main interview questions and their order are mostly fixed. While many chatbot systems lacked empathetic utterances found in daily dialogue and utterances related to the bot’s consistent experience, Blender (Smith et al., 2020) could generate utterances those appear in a daily dialogue by using a huge database of dialogues, which incudes emotional utterances and utterances influenced by a speaker’s personality, for end-to-end learning. However, it is still difficult to continue the dialogue for a long turn.

### 3.7 Comparison of the existing systems

Kopp et al. (2005, 2006) developed the conversational agent Max that could proactively interact with users based on the agent’s internal goal and intention (the behaviors were generated using the BDI model). The system manages the dialogue with three control layers to deal with the start/end of dialogue and turn-taking control. It also has the emotional system to express the agent’s emotion (Becker and Wachsmuth, 2010). The agent Max is equipped with many of the functions necessary for a daily dialogue though the target of the agent is not a daily dialogue for gaining understanding and empathy with others. This section describes some functions needed for a daily dialogue; however, no study has integrated all of these functions into a dialogue system, which is the target of this study.

## 4 Development of dialogue system

### 4.1 Policy of dialogue management

**FIGURE 1:**
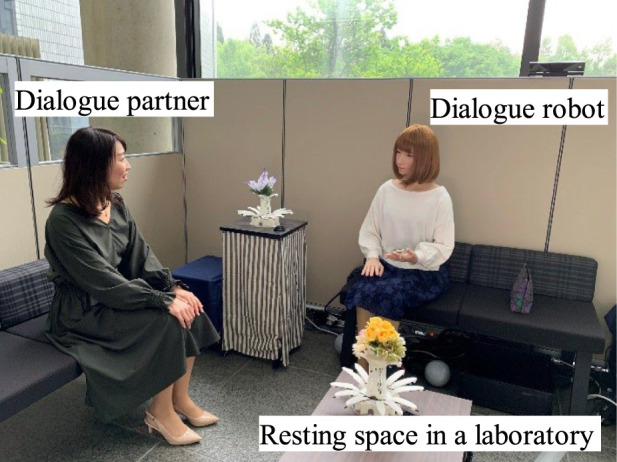
Target robot and dialogue situation in this study.

This study assumes the situation where the robot talks with a stranger for about 10–20 min (first-meeting dialogue). We assume this certain situation since it is difficult to design the robot’s desire in an open context. More concretely, the robot is in a resting space in a laboratory and talks with a person who visits the laboratory (Japanese conversation is assumed) as shown in Figure 1.

The developed system in this study automatically selects a short dialogue script designed by human designers based on the robot’s internal state or observed information (sensor input) to realize daily dialogue. The system consists of a dialogue script of a certain length in a topic, the robot’s internal state, the internal state of the dialogue partner (estimated), and the memory about the dialogue.

### 4.2 Script

**FIGURE 2:**
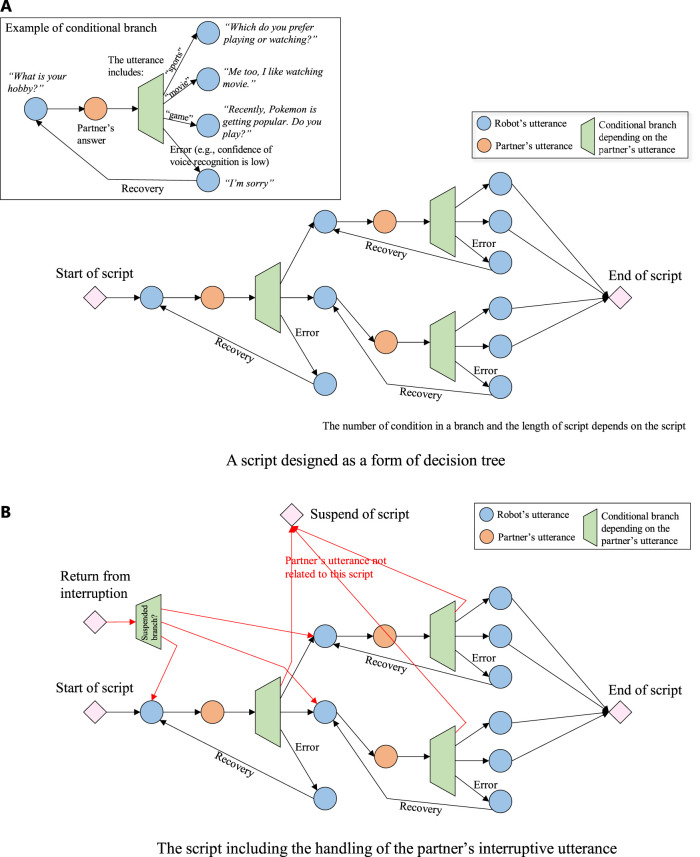
Concept of script. **(A)** A script designed as a form of decision tree. **(B)** The script including the handling of the partner’s interruptive utterance.

The script is described as the utterances of the robot and the expected responses of the person (dialogue partner) in the manner of a decision tree. The robot waits for the utterance of the partner and selects its utterance by following the decision tree along with the partner’s utterance. The contents to be spoken are defined in advance in the script. Although the scripts are predefined, the robot can refer to the memory of the past dialogue and repeat the partner’s information such as name and age which were spoken by the partner. In the developed system, a script is designed in the form of decision tree as shown in Figure 2A.

**FIGURE 3:**
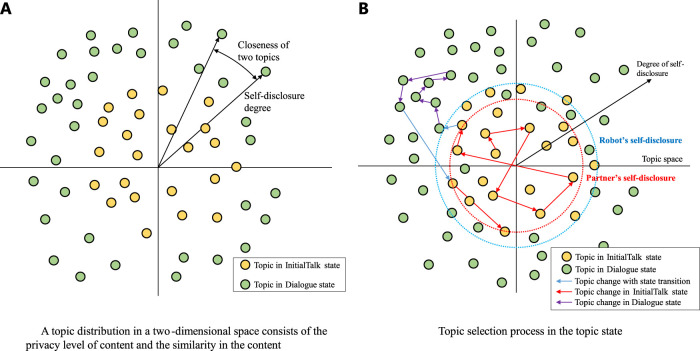
A topic distribution in a two-dimensional space. **(A)** A topic distribution in a two-dimensional space consists of the privacy level of content and the similarity in the content. **(B)** Topic selection process in the topic state.

The script is designed to have a topic (a type of dialogue content, such as favorite food, event in the weekend, and trouble at work). The topic has the two-dimensional feature to express privacy [degree of self-disclosure (Jourard 1971)] of content and type of content. This feature space is defined in a polar coordination, and the distance from the origin represents the degree of self-disclosure of the topic as shown in Figure 3A. The angular position represents the type of content, and the angle between the topics represents the relative closeness of their contents (Therefore, the absolute angular position has no meaning).

### 4.3 Design of desire for daily dialogue

To design the robot’s desire and intention in a daily dialogue of a first meeting, we referred to the studies of human–human conversations. The politeness theory (Brown and Levinson, 1987) says that people basically have a desire for approval and acceptance by others (positive face) and a desire to proceed without being impeded upon (negative face). It is reasonable that the robot has a desire for approval (self-esteem) to gain self-worth. The robot should make a good relationship with the dialogue partner in order to be approved of and, at a same time, follow social norms to be accepted by others. To build a good relationship with the other party in the first-meeting dialogue, the robot requires to know whether the dialogue partner likes the robot (favorability to the robot) and it has a favorability toward him (favorability from the robot). By estimating the relationship between the robot and the partner based on their favorability, the robot can determine a topic to talk about which has the degree of self-disclosure allowed owing to their relationship. This has also been suggested by the uncertainty reduction theory (Berger and Calabrese, 1975) which explains that people try to know their partner in order to decrease uncertainty and increase the predictability of his/her behavior. They make a self-disclosure to reduce the uncertainty of each other (Svennevig, 1999). The breadth and depth of self-disclosure increases as their relationships move toward intimacy as suggested by the social penetration theory (Altman and Taylor 1973), which suggests that disclosure will be maximal in the early stage of a relationship.

Such a balancing behavior, or reciprocal behavior, has been investigated from the perspective of evolutionary psychology and neuroscience. From an evolutionary psychological perspective, reciprocal behavior in humans is a necessary selection for adaptation to the environment (Trivers, 1971). It has been reported that reciprocal behavior has also been observed in primates (Silk, 1987). Human cognitive characteristics have also been investigated in terms of reciprocal behavior, e.g., a bias that people have in unconsciously remembering the faces of traitors to maintain a reciprocal relationship (Oda and Nakajima, 2010). Neuroscience has also shown that altruistic behavior alleviates physical pain. The role of networking in brain regions associated with the tendency to make altruistic decisions has also been highlighted. Izuma et al. (2008) experimentally demonstrated that the striatum, which is believed to be involved in the reward system, is activated in the brain when a person receives a good reputation from others, and it has been neurophysiologically demonstrated that receiving a good reputation works as a reward. Such reciprocity, which is seen in primates and humans, is essential for robots to behave in a manner that allows natural interaction.

To follow the social norms in the first-meeting dialogue, the robot should behave by keeping up reciprocity, that is, the robot should behave for not only itself but also the partner. Psychological studies have shown that people have a good impression of the conversation partner when the amount of speech exchange is balanced (Ogawa, 2000).

**FIGURE 4:**
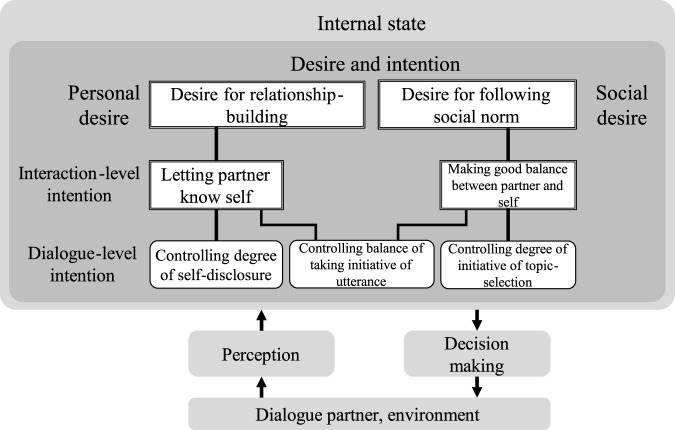
Principle for decision-making of the robot in the first-meeting dialogue.

Based on the aforementioned knowledge, we designed the desire–intention–behavior structure as shown in Figure 4. The lines mean relationships between the desires and intentions. Multiple desires and intentions affect the decision-making in which the robot chooses a script during the dialogue. This works as a principle to design the scripts for the first-meeting dialogue. The robot's responses and utterances are designed to follow these desires and intentions.

### 4.4 Internal state of the robot

To develop a robot that behaves according to the desires designed as mentioned above, we define the internal state of the robot involved in the behavior that satisfies these desires. For personal desires, the robot controls its behavior such that the relationship with the dialogue partner becomes the desired one. To be more specific, the robot behaves in such a way that the higher the robot’s favorability toward the partner, the deeper the relationship with the partner. The relationship refers to the degree of self-disclosure. In other words, it is judged that the relationship has deepened when the degree of self-disclosure is high not only for one partner but also for each other. Therefore, we define the internal states of the robot’s favorability and self-disclosure. As will be described later, the favorability of the partner in the dialogue depends on whether or not the interests in the topic match each other.

For social desires, the robot recognizes the partner’s favorability toward the robot and controls the balance of the initiative of the dialogue such that it increases. In addition, as aforementioned, the emotional expression of the robot is important in daily dialogue. It is assumed that the robot has emotions related to the degree of interest in the topic and the favorability of the partner. We also assume that the emotion influences the decision-making of the robot.

**FIGURE 5:**
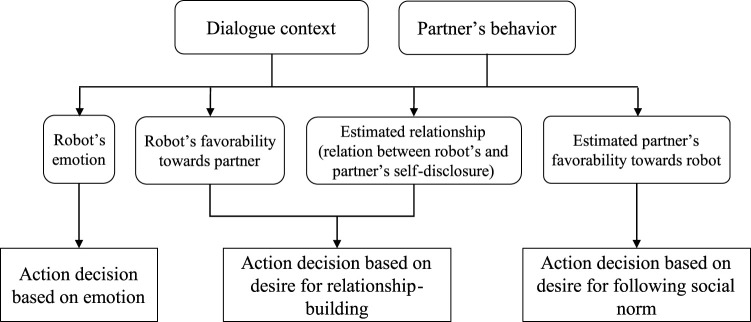
Relationship between the internal states and desires of the robot.

Summarizing the above, the relationship between the internal states and desires is as shown in Figure 5. The following describes each internal state.

### 4.5 Feeling state

To represent the robot’s emotional state, the PAD (Pleasure, Arousal, Dominance) model (Mehrabian, 1996), which is easy to correspond to the features of facial expressions and movements, is adopted. The FACS model (Ekman, 1999) represents more complex emotions, but it is specific to facial expressions. To design an internal model of the robot, which is related to decision-making, the basic emotional model is used. In this system, two types of components are separately controlled. One changes depending on the response of the dialogue partner in short term, and the other gradually changes through the dialogue. The study indicates the former as immediate*-*emotion and the latter as mood. Each of immediate-emotion and mood has three dimensions (pleasure, arousal, and dominance). Each dimension ranges from −1.0 to 1.0. The immediate-emotion change is defined according to the branching in the decision tree of the script. The mood gradually changes over time, owing to the facial expressions of the partner. The update rule of pleasure dimension in the mood is as follows: 1) decrease in attenuation coefficient by 0.8 every minute. 2) Synchronize with the pleasure level of the partner. 3) Deteriorate when the dialogue breaks down. 4) Increase or decrease according to the degree of empathy when the topic changes (see Table 1). The dimension of arousal in the mood is updated as inversely proportional to the relationship to express tension and relaxation, such that the lower the relationship, the higher the arousal with becoming tense, and the higher the relationship, the lower the arousal with being relaxed. The dominance dimension in the mood is proportional to the self–other priority (described later) to express the dominant state when the robot prioritizes itself.

**TABLE 1:** Robot’s degree of empathy owing to the attitude of the robot and partner.

Robot	Partner	Degree of empathy
Favorable	Favorable	1.0
Favorable	Neutral	0.5
Favorable	Unfavorable	−1.0
Neutral	Favorable	0.5
Neutral	Neutral	0.25
Neutral	Unfavorable	−0.5
Unfavorable	Favorable	−1.0
Unfavorable	Neutral	−0.5
Unfavorable	Unfavorable	−1.0

Next, we will explain the rule of the integration of immediate-emotion and mood into the robot’s emotional state. Since immediate-emotion is a short-term expression which changes owing to the partner’s utterance, the emotion immediately follows the immediate-emotion when it changes. If the immediate-emotion is not expressed, the emotion follows the mood. When the emotion follows the immediate-emotion, the emotion changes at a rate depending on the current emotion (fast change when there is high arousal in the current emotion and gradual change when there is low arousal in the current emotion). When the emotion follows the mood, it gradually shifts to the mood.

For the update rule (2) of the pleasure dimension in mood described above, the partner’s facial expression captured by a camera is recognized every 50 msec Then, the ratio of the facial expressions during when she is speaking in a topic is calculated. The facial expressions are categorized into three groups; happy and surprise are counted as positive facial expressions, sad and angry are counted as negative facial expressions, and the others are counted as neutral facial expressions. Here, let *f*_*p*_, *f*_*nu*_, and *f*_*ng*_ be the ratios of positive, neutral, and negative facial expressions, respectively. The pleasure of the robot’s mood is reduced by 0.03*f*_*ng*_ and increased by 0.03*f*_*p*_ to implement the attraction to the facial expression of the dialogue partner.

The robot estimates the partner’s pleasure of mood (only the pleasure dimension is estimated) from the facial expression. The estimated pleasure is reduced by 0.05*f*_*ng*_ and increased by 0.05*f*_*p*_.

### 4.6 Interest in the topic

We assume that the robot has different levels of interest in the topics. Here, we assume three levels: favorable, unfavorable, and neutral. The robot’s interest in each topic is manually defined in advance, for example, we define that the robot likes a topic about romance novels and does not like a topic about having a meal.

The robot estimates the partner’s degree of interest in the topic on which they are talking about based on their facial expressions. If the ratio of the neutral face (*f*_*nu*_) is larger than the other facial expressions (*f*_*p*_ and *f*_*ng*_), then the interest is recognized as neutral. In other cases, if a happy or surprised face is recognized more than a sad or angry face, the interest is estimated to be favorable. Otherwise, the interest is recognized as unfavorable.

### 4.7 Empathy

The robot has an empathy with the dialogue partner based on their interests in the topic. Each time a topic is over, the robot compares its interest with the estimated partner’s interest and determines the degree of empathy as shown in Table 1. The degree of empathy ranges from −1.0 to 1.0. The empathy is basically increased as both interests in the topic match, but in the case of unfavorable, the empathy has a negative value even if both interests match since it is unpleasant to talk about a topic that the other dislikes.

There are two types of empathy: cognitive empathy and emotional empathy (affective empathy). The former is the ability to understand how a person feels and what they might be thinking. The latter is to share the feelings of another person, and it requires the estimation of psychological logic and emotion and adaptation to the other person. The empathy in the proposed system is related to cognitive empathy rather than emotional empathy.

### 4.8 Favorability toward the dialogue partner

The robot’s favorability toward the dialogue partner affects the topic selection. When the favorability is high, it talks about a topic which seems to be favored by the partner to continue the dialogue. The favorability ranges from −1.0 to 1.0. It changes with three events. The first is when the partner gives the answer that the robot prefers in the dialogue. For example, if the robot asks for a genre of favorite book and it likes romance, the favorability is increased only when the partner's answer is romance. The second is when a dialogue breakdown (when the robot cannot recognize the partner’s speech or when speech collision occurs) is detected. The favorability is decreased since it is difficult to talk well with a person who makes the dialogue break down. The third is when each time a topic is over. At the end of the topic, the favorability is changed according to the empathy as shown in Table 2.

**TABLE 2:** Robot’s favorability change owing to both robot and partner’s interest in the current topic.

Robot	Partner	Change of robot’s internal state
Favorable	Favorable	Mood (pleasure) +0.1
		Favorability +0.1
Favorable	Neutral	Mood (pleasure) +0.05
		Favorability +0.05
Favorable	Unfavorable	Mood (pleasure) −0.1
		Favorability −0.1
Neutral	Favorable	Mood (pleasure) +0.05
Neutral	Neutral	No change
Neutral	Unfavorable	Mood (pleasure) −0.05
Unfavorable	Favorable	Mood (pleasure) −0.1
		Favorability −0.1
Unfavorable	Neutral	Mood (pleasure) −0.05
		Favorability −0.05
Unfavorable	Unfavorable	Mood (pleasure) +0.1
		Favorability +0.1

### 4.9 Estimated partner’s favorability toward the robot

The robot estimates how the partner favors itself through the partner’s speech and behavior.

1) Estimation based on the speech
  When the robot detects compliments each time a voice recognition result is acquired, it updates the favorability by increasing it by 0.05.
  Every time the voice recognition result is acquired, it is judged whether it is a question or not, and in the case of a question, 0.02 is increased. This means that the robot guesses that the partner asks it because she has an interest with it.
  The robot compares the amount of speech between the partner and itself every 50 s. If the partner’s speech is more than the robot’s speech, the favorability is increased by 0.02. This means that the robot guesses that the partner speaks much since she has an interest in the robot.
  If the robot detects a contemptuous phrase in the partner’s speech, the favorability is decreased by 0.2.
  When the partner ignores the robot’s question and does not answer, the favorability is decreased by 0.05.
2) Estimation based on the behavior
  The robot recognizes whether the partner looks at it by the facial image every 50 msec Every 50 s the favorability is increased by 0.02 if the partner is looking at the robot longer than not looking.
  The favorability is decreased by 0.02 every 50 s if the partner spends more time looking in a completely different direction than looking at the robot.

### 4.10 Self-disclosure

The degree of self-disclosure indicates how much private talk is allowed with the dialogue partner. It takes a value of 0.0–1.0. This value is on the same scale as the self-disclosure of the topic. The candidates of topic selection are determined by referring to either of the robot’s and partner’s self-disclosure degree (described later).

The degree of the robot’s self-disclosure basically follows the favorability toward the partner. This means that the robot tries to self-disclose more deeply and deepen the relationship with the partner with whom it has high favorability. On the other hand, if the robot has low favorability toward the partner, the degree of self-disclosure decreases, and the selectable topics are reduced. This leads to the ending of the dialogue without self-disclosure. The degree of self-disclosure is updated every time the topic is changed.

The robot estimates the partner’s self-disclosure based on how much the partner talks about specific matters. For example, if the partner answers “I’m from XX street” rather than “I’m from YY city” when the robot asks the partner’s hometown, it estimates that he/she self-discloses more deeply. The specificity is measured by the term frequency–inverse document frequency (TF-IDF; Rajaraman and Ullman, 2011). The estimated self-disclosure degree of the partner becomes larger if the specificity is large and the self-disclosure degree of the current topic is large. The estimation equation is defined as follows:

$${SD}_{p}=\left(a\;t_{f}+b\right)\;{SD}_{t}.$$

where *SD*_*p*_ is the estimated partner’s self-disclosure degree, *SD*_*t*_ is the self-disclosure degree of the current topic, and *t*_*f*_ is the specificity of the word in the partner’s utterance measured by TF-IDF (*a* and *b* are predefined constants tuned by the system designer).

### 4.11 Relationship

The robot recognizes that the relationship with the partner is strong if both self-disclosure degrees are large. The strength of the relationship is calculated as the average of both self-disclosure degrees. By referring to the estimated relationship, the robot behaves while balancing both the self-disclosure degrees. For example, when the robot’s self-disclosure degree is high, it selects a topic with a high self-disclosure degree. But this behavior might not be suitable if the partner’s self-disclosure degree is not high, especially for the situation of the first-meeting dialogue in a public space. The robot can avoid such a behavior by referring to the relationship.

The robot changes its speech style in three levels according to the degree of intimacy with the partner and social status of the partner. The intimacy degree refers to the relationship strength described above. The social status (superior, subordinate, close, distant) is recognized in the contents of dialogue (the robot asks the partner’s social status in some scripts). If the speech level is low, the robot’s speech is honorific. If it is high, the robot’s speech is broken in tone.

### 4.12 Degree of giving priority to partner

The self–other priority takes from −1.0 (giving priority to the partner) to 1.0 (giving priority to self) in the robot’s internal state that determines whether the partner’s action is prioritized or the robot’s own desire is prioritized when the topic changes. The robot recognizes the ratio of the recent eight topics that are selected by the partner. If the ratio is much biased to the partner (the partner mainly took an initiative to select the topics), it increases the self–other priority such that the partner continually takes an initiative in the topic selection to maintain the dialogue context and *vice versa*. More concretely, if the robot mainly selects the topics in the recent dialogue, the priority is increased by 0.1, and if the partner mainly selects the topics, the priority is decreased by 0.1.

In addition, the robot also balances the initiative of topic selection between the robot and partner to follow the law of reciprocity. The priority is decreased by 0.1 if the robot mainly took the initiative (selected the topics) so far and *vice versa*.

### 4.13 Memory of dialogue

In this system, the robot memorizes the partner’s personal information (e.g., name, age, and favorite food), topics the robot talked about, and the partner’s answer to the robot’s question. The memory information can be accessed from the script and used for decision-making. For example, when the robot talks with the partner for the second time, it does not repeat what it talked with the person in the first time.

**FIGURE 6:**
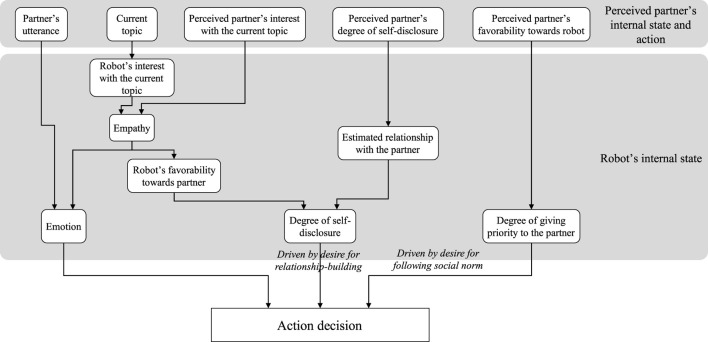
Implementation of the robot’s action decision based on its desire in the first-meeting dialogue.

Figure 6 summarizes how the robot’s decision-making is implemented following the intentions and desires in the first-meeting dialogue shown in Figure 4. It also summarizes how the partner’s behavior influences the robot’s internal state.

## 5 Dialogue management

### 5.1 Dialogue state management

The dialogue management system automatically selects the topics based on the internal state. There are two types of topic selection: when the robot voluntarily selects the topic (Active) and when the dialogue partner asks a question, and the robot selects a question-related topic (Passive) (In Passive selection, it can be said that the partner selects the topic.) The management system discriminates the dialogue state by how the current topic is selected (i.e., it manages who took the initiative to decide the current topic). In addition, the system deals with a state where nobody is speaking. Here, the sequence state is defined by the following three states:

1) Silence: a state between topics (silence after a topic is over),
2) Active: a state where the robot has selected a topic, and
3) Passive: a state where the partner has selected a topic.

Basically, the dialogue control routine is to wait for a certain period when the Silence state starts, and if the partner starts to talk during the Silence state, the appropriate topic is selected, the sequence state is moved to the passive state, and the robot starts the script related to the selected topic. Alternatively, when the waiting time has expired, the robot selects a topic to continue the dialogue, moves to the active state, and starts the script related to the selected topic. The process when the partner starts a new topic while another topic is being talked (during active or passive state) is called interrupt process. To make the topic selection strategy depend on a dialogue situation, the following five interaction states are defined and the different topic strategies are implemented:

1) Idle: a state where a dialogue has not started (no one has started to talk).
2) InitialTalk: a state in which a dialogue begins and the robot and partner talk about topics with a low degree of self-disclosure (shallow talk such as sharing personal information).
3) Dialogue: a state where the robot and partner talk about topics with a high degree of self-disclosure (deep talk such as sharing private information and empathizing with each other rather than merely sharing information).
  Exception: an error state such as being unable to understand what the other has said. Exceptions include two types.
  NoMatch Exception: a state where there is no selectable topic that matches with the speech of the partner.
4) NoSelectableTopic Exception: a state where there are no more selectable topics (for example, all predefined scripts are already talked about).
5) Close: a state where the dialogue is completed.

Each topic is assigned to one sequence state and on interaction state. For example, the topic of “asking the partner’s job” is assigned to Active and InitialTalk state, and “answering the partner’s question to ask the robot’s hobby” is assigned to the Passive and Dialogue states. The topics assigned to the Passive state can be selected when the partner asks a question to the robot.

**FIGURE 7:**
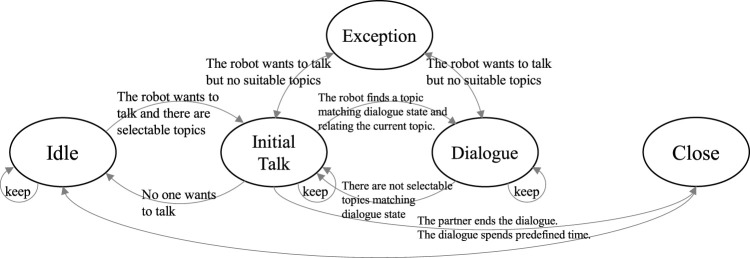
Transition of interaction states in the first-meeting dialogue.

Figure 7 shows the constraint on the transition between the interaction states. The transition rule is as follows:

1) From all states: when a Passive topic is selected, the interaction state transfers to the one to which the topic belongs.
2) To all states: accept a control that forces a transition within the topic.
3) From Idle to InitalTalk: when the robot finds a person in the Idle state (the topic “starting talk to a person” is selected).
4) From InitalTalk to Dialogue: when the robot spends a certain period in InitialTalk state and there are selectable Active and Dialogue topics related to the current topic.
5) From Dialogue to InitalTalk: when the robot tries to select an Active topic but there is no selectable Active topic. In addition, when there is no interaction for a certain period in Dialogue state.
6) From InitialTalk to Idle: when the robot tries to select an Active topic but there are no selectable Active topics, and the partner is not looking at the robot.
7) From InitialTalk or Dialogue to Exception: when there are no more topics that can be selected while the robot tries to select an Active topic, the state transfers to Exception (NoSelectableTopic Exception). The state transfers to Exception (NoMatch Exception) when no topic matches the partner’s utterance in Silence state.
8) From Exception to InitialTalk or Dialogue: when the exception-handling script (described later) ends, the state returns to the previous state.
9) From InitialTalk to Close: when the dialogue partner ends the dialogue (e.g., saying good-bye) or the dialogue spends predefined time (e.g., 15 min), the state transfers to Close, and the robot tries to end the dialogue.
10) From Close to Idle: when the robot detects that there is nobody to talk to.

### 5.2 Wait in Silence state

As described above, the robot waits for a certain period after a script is over. To adapt to the partner’s speech rhythm (how much the partner usually has a latency before starting to talk), the robot calculates the average turn-taking time in the past and basically waits for that time. In addition, the robot shortens the waiting time when the robot prioritizes itself according to the self–other priority. If the robot prioritizes the partner, the robot makes the waiting time longer such that it waits for the partner’s speech. The waiting time *T*_*w*_ in the Silence state is calculated as below:

$$T_{w}=T_{h}-C_{r}S_{r}.$$ (1)

where *T*_*h*_ is the time duration between the onset of the Silence state and the onset of the partner speech in the last Silence state, and *S*_*r*_ is the degree of self–other priority. *C*_*r*_ is a constant parameter. As shown in the equation, the higher the self–other priority is, the shorter the waiting time gets.

### 5.3 Interrupt process

When the robot in the Active state recognizes the partner’s utterance, it checks whether there are Passive topics that are suitable to respond to the partner’s utterance. If there is such a topic (here, it is called “new topic”), the interrupt process starts. It consists of three types of interruption manners and two types of return manners:

1) Interruption manner:
  Interrupt: stop the current script and shift to the script of a new topic (the partner is prioritized in this case).
  Stack: suspend the current script, state that the robot continues the current script, and start a script of a new topic after the current script ends (the partner is prioritized in this case).
  Ignore: ignore the interruption and start a script of a new topic after the current script ends (the robot is prioritized in this case).
2) Return manner:
  Back: return to the original script that was interrupted.
  Skip: transfer to the Silence state without returning to the original script.

**FIGURE 8:**
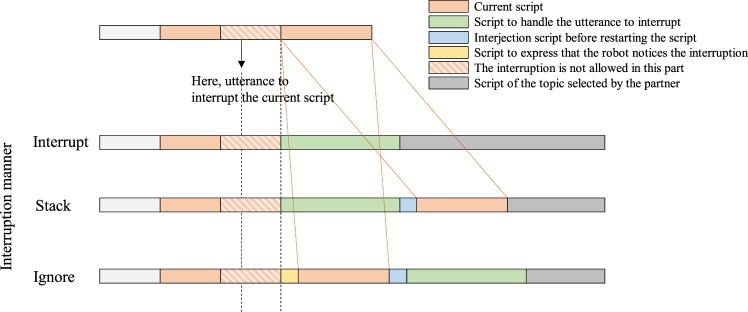
Pattern of handling the partner’s interruptive utterance.

The pattern of handling interruption is shown in Figure 8. The importance is to let the partner know that the robot is properly recognizing the interruption by saying something like “I will talk about it later” when the Stack or Ignore manner is used.

**FIGURE 9:**
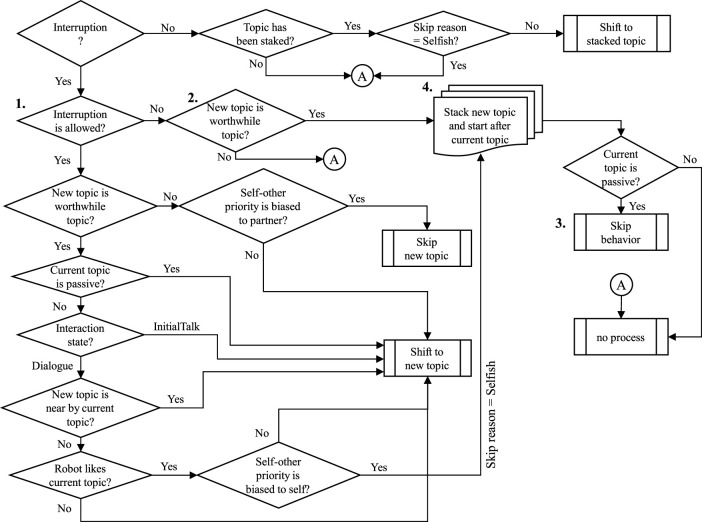
Flowchart of the interrupt process.

The flowchart of the interrupt process is shown in Figure 9. At condition 1 in the flowchart, if a new topic must be talked about immediately (this kind of topic is called as worthwhile topic) or if the current topic allows for interruption and the script does not partially inhibit the interruption, the current topic is stopped and a new topic is started. At condition 2, if the interruption is inhibited, the robot suspends starting a new topic until the inhibition is cleared. Then the robot can quickly start a new topic. At process 3, when the robot ignores the interruption, the robot shows a skip behavior to let the partner know that the robot understands that the partner talks about a new topic. At process 4, the robot stacks a new topic in the stack memory and continues the current topic (this means that the robot prioritizes itself). It starts the stacked topic after the current topic is over. At the beginning of a new topic, the robot says words that reminds the partner of the topic, such as “I’d like to answer the question you asked some time before ....” The example of script including the interruption process is shown in Figure 2B.

### 5.4 Strategy of topic selection

To maintain the dialogue context, the topic selection is controlled by the distance on the topic space consisting of the self-disclosure degree (radial direction) and closeness of the topic content (circumferential direction) (as shown in Figure 3B). If the robot’s self–other priority is high, it gives itself the priority and chooses a topic that falls within a certain range around its self-disclosure degree. When giving a priority to the dialogue partner, topics within a certain range around the estimated self-disclosure degree of the partner is selected.

The robot in the InitialTalk state should select topics to share information widely with the partner, and the circumferential range is not limited. But, if the interest of the dialogue partner to the current topic is high, the robot selects a topic closest to the current topic. Otherwise, the selectable topics are sorted in order of the distance from the current topic in the predefined range and the topic with the median distance is selected. In other words, if the partner is not strongly interested in the current topic, the robot selects a topic with a content different from the current topic to some extent. This enables the robot to explore the topic in which the partner has interest (the red arrows in the figure are examples of topic transition in the InitialTalk state).

The topic selection in the Dialogue state is to elaborate the current topic. The selectable topics' area is near the area of the topic that has been selected so far. If the interest of the partner to the current topic is low, the robot selects the topic with the median distance from the current topic. Otherwise, it selects the topic closest to the current topic. In other words, unless the partner is not interested in the current topic, the topic to elaborate the current topic is selected (the purple arrows in the figure are examples of topic transition in the Dialogue state).

In the Idle state, the robot does not actively start to talk since there is no dialogue partner. In the Exception state, one topic is prepared to handle NoMatch Exception and another topic is prepared to handle NoSelectableTopic Exception. In the Close state, only a topic for farewell greetings is selected. Therefore, a topic can be uniquely selected in the states other than the InitialTalk and Dialogue.

To handle NoMatch Exception (no topic is related to the partner’s utterance), the robot extracts nouns from the partner’s utterance and searches for relevant Active topics by referring word2vec distance (Mikolov et al., 2013) between the noun and topic subject (e.g., “name,” “favorite food,” “weekend”). Only if the partner’s utterance is a question, does the robot simply answer it. Then, the robot starts the topic after saying a filler phrase. Otherwise, the robot says, “I don’t know what to do,” “I’m sorry I don’t know what to talk,” looking down to show it has some trouble. To handle NoSelectableTopic Exception, the robot makes a statement to end the dialogue, such as “it's about time”, and the state transfers to Close.

**FIGURE 10:**
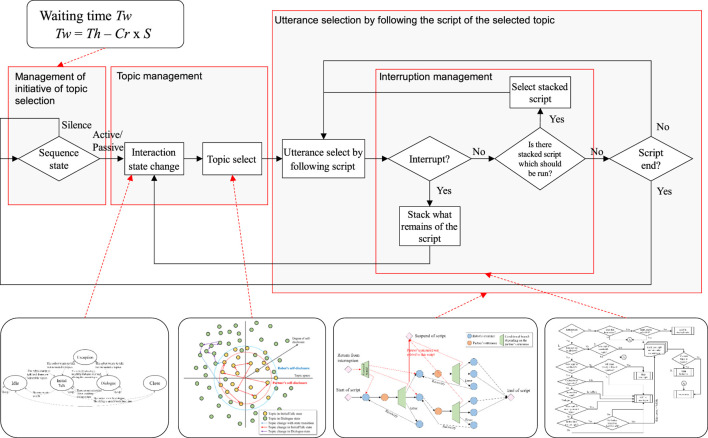
Block diagram of dialogue management.

The total dialogue flowchart which includes the interruption process is shown in Figure 10. The dialogue flow is roughly divided into three parts: the management of initiative of topic selection (who took initiative to select the current topic), the management of topic selection, and the utterance selection by following the script of the selected topic. In addition, the interruption process is managed in the script. Equation 1 that determines the waiting time after the end of the last script is related to the initiative management part. The interaction state transition (Figure 7) occurs before topic selection, and the robot selects the topic by following the process shown in Figure 3B. The robot chooses utterances by following the script of the selected topic, as described in the decision tree form shown in Figure 2. During the script, the robot manages an interruptive utterance of the partner when it occurs, as shown in Figures 8, 9.

### 5.5 Movement design of robot

By linking the internal state not only to language but also to nonverbal expressions such as facial expressions, people can feel that they are naturally interacting with the robot, which is not felt during a text chat. The Emotion described above appears in the facial expression of the robot. In addition, since the more intimate the people are, the more they tend to turn their bodies toward the other party (Argyle and Dean, 1965), the orientation and tilt of the robot’s body are controlled according to the robot’s favorability toward the dialogue partner. The arm movement such as pointing gestures and depicting gestures are closely related to the utterance. The script designer designes those gestures in advance and defines the onset timing of the gestures to synchronize them with the robot’s utterance in the script. In addition, subconscious movements such as blinking, gaze averting, and lip movement associated with utterances are automatically generated.

## 6 System evaluation

### 6.1 Dialogue by proposed system

We implemented the dialogue system and scripts to realize daily dialogue in the first meeting in a lounge space. Specifically, there is a table with sofas in the lounge space and the android robot is sitting there. A subject who comes to the space for resting has a dialogue with the android. We created scripts that assume the dialogue in this situation. As for the contents, we created 80 scripts for 25 topics such as where the subject came from, about whether they are interested in robots, and about their favorite books. To handle Active and Passive sequence states, we prepared two types of script for the same topic: one is the script that the android initiates the topic and the other is the script that the dialogue partner initiates the topic.

In this situation, it is not necessary for the partners to proactively talk to the android and keep talking with it. In other words, there is no clear objective of the conversation. The conversation process depends on the person’s personality and mental state. When we tested the developed system, we observed some patterns of the relationship shift such as those below:

1) The android and the person gradually disclosed themselves (the android chose the scripts from shallow to deep levels and the person gradually increased the amount of speech) and built a relationship in which both exchange their names.
2) The android’s self-disclosure level became significantly different from the person’s level in the conversation. Then, the android reciprocally chose the scripts which fit to its level and the scripts which fit to the person’s level.
3) In the beginning, the android proactively talked to the person, but he merely made short responses and started to use his smartphone. Then, it did not proactively talk, and both did not talk to each other.

There still remained dialogue breakdown that could not be recovered by the developed system. But the resultant interaction flow and the developmental process of the relationship were reasonable and natural. The conversation control based on the android's desire and intention might produce natural interactions.

### 6.2 Android robot

The android ERICA (shown on the right of Figure 1) has a very humanlike appearance and 44 degrees of freedom in the upper torso that enables humanlike gaze behaviors, lip and head motions, facial expressions, and bodily gestures. The text-to-speech software (provided by HOYA Corporation) provides humanlike synthesized voice. The sensor network consists of depth sensors, microphone arrays, and cameras that can detect and pursue multiple persons’ positions, estimate speaking persons, recognize their voices, and recognize facial expressions (Glas et al., 2016). When the android speaks, it moves its lips, head, and torso in synchronization with the prosodic features of its voice. Those movements are automatically generated based on an existing system developed by Ishi et al. (2012) and Sakai et al. (2016).

### 6.3 Multimodal Turing test

The developed android is limited to the situation of dialogue in the first meeting, but it is possible to have daily dialogues with people for about 10 min using various expression modalities. While various methods can be considered as the method for evaluating this performance, the total Turing test (Harnad, 1991) can be considered as a method for comprehensively evaluating the naturalness of the dialogue. However, the robot developed in this research specializes in dialogue, not object manipulation by the robot. Therefore, in this study, we evaluate the naturalness of the dialogue by a test to see if the autonomous dialogue android is indistinguishable from the android that is being remotely controlled by a person. If the subject who has a talk with the android judges that the android is remotely controlled by a human, this means he/she thinks that the android talks in a humanlike manner. On the other hand, if he/she judges that the android behaves autonomously (i.e., the android is fully controlled by a computer program), this means that he/she feels unnaturalness in the android behavior, for example, when the android makes some mistakes which humans never make. We call this the multimodal Turing test (MTT).

**FIGURE 11:**
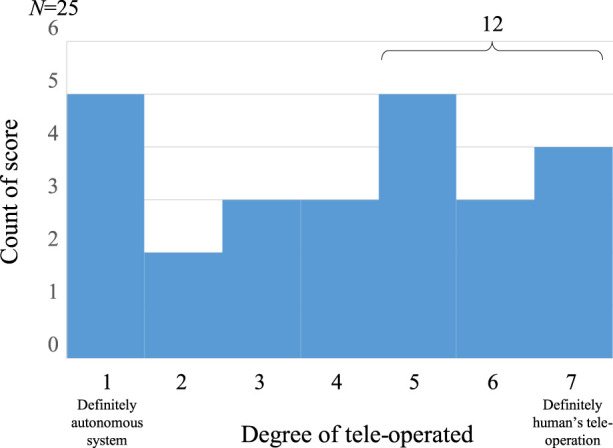
Result of MTT.

We conducted an experiment to evaluate the developed android with MTT. As an experimental procedure, we first told the subjects that “this android works in two ways. One way is that the android behaves autonomously (autonomous mode) and the other way is that it behaves by remote control (teleoperation mode),” and asked them to talk with the android without telling which mode they were experiencing (actually, all subjects interacted with the android in the autonomous mode). Then, after the dialogue, we let them evaluate how much they thought that the android was remotely controlled (7 scales). As a result of the evaluation with 25 subjects [average 46.0 (std. 12.3) years old], several subjects answered that they thought they were interacting with an android in teleoperating mode (the result is shown in Figure 11). To be more concrete, 12 of the 25 subjects scored more than 5 points, which means that they felt the android was remotely controlled to some degree. On the other hand, there were some subjects who answered that the robot was in the autonomous mode. From the interviews with them, it is thought that the dialogue breakdown on turn-taking and the timing of the android’s backchannel were wrong which led to that decision.

By introducing script-based dialogue and desire-based dialogue management, it was possible to reduce the breakdown of the dialogue context, but the breakdown in turn-taking has a great influence on the evaluation in this experiment. In the existing chatbot systems, not much attention has been paid to the importance of turn-taking in a dialogue because turn-taking is systematically guaranteed due to text input, but from the results of this experiment, it is speculated that smooth turn-taking in multimodal dialogue cannot be simply realized and the breakdown in turn-taking results in an unnatural (not humanlike) impression of the dialogue. Nonverbal communication has been shown to account for a large proportion of communication (Mehrabian and Weiner, 1967). To pass the MTT, it is therefore necessary for us to deeply understand natural nonverbal interaction. The developed android could make people think that it was operated by a human (e.g., it behaved in a humanlike manner) in the limited situation in this study.

## 7 Discussion

### 7.1 Dialogue control architecture

The existing chatbot systems continue a dialogue by selecting or generating an utterance that is a semantically correct response to the partner’s utterances, but the system does not have a motivation to keep the context underlying the background of the dialogue. Therefore, in many systems, the dialogue easily breaks down in about 10 turns. If we predefine a dialogue script and the robot follows it, it is possible to keep the consistency of the context. But the predefined story is not suitable for daily dialogue and the topic in the dialogue should be freely chosen depending on the dialogue situation. The predefined script does not allow the robot to respond to the partner’s utterance that is not assumed in the script, such as switching to the side sequence. Therefore, the robot should be able to switch the current topic during the script. In addition, unlike discussions and interviews, in daily dialogue, the timing of turn-taking cannot be clearly determined. To realize smooth turn-taking, the robot needs to judge the end of the partner’s turn at every end of the partner’s voice activity. Through the implementation of multimodal daily dialogue, it turns out that it is necessary to make action decisions at three levels—that is, dialogue state decision, script decision, and turn-taking decision—to continue a natural dialogue without breaking the context, and furthermore, it is necessary to design the evaluation function for those decisions. It can also be seen that in the non–task-oriented dialogue, those evaluation functions are determined by the robot’s desires which determine the behavior policy.

This study, on the other hand, has not focused much on the architecture of motion generation of the robot. The Situation, Agent, Intention, Behavior, Animation (SAIBA) (Kopp et al., 2006) is a unified framework to generate multimodal expressions of agents. In this framework, the Behavior Markup Language (BML) contributes to bridge the agent intention and its behavior generation. We are also developing the android motion generation system which is helpful to describe the motions those appear in a daily dialogue.

### 7.2 Contents of daily dialogue

A daily dialogue does not have a clear purpose, and the content of the dialogue itself does not matter if a relationship with the other person can be built through understanding and empathy. A dialogue system based on a big database is desirable for an open domain dialogue, but it is still difficult to continue a dialogue while maintaining context in existing systems. Therefore, the developed system focuses on continuing a dialogue while maintaining the context. We think it is possible to have a daily dialogue even if the story of the dialogue is limited to some extent. A designer creates scripts consisting of a few to a dozen turns of utterance exchanges in advance and gives them to the robot as candidates of topic selection. In the experiment, the participants could continue the dialogue without dissatisfaction even if the story of the dialogue was decided in advance. (Since the robot can respond to a question by giving a simple answer, it can avoid a context failure with the minimum response even if the participant asks a question that deviates from the designed story.) In daily dialogue, people talk about anything rather than something that they want to talk about clearly. People expect an interaction that connects short topics, even though a story is limited to some extent.

However, the limited number of predefined scripts reduces the scalability of the system. The architecture that consists of a set of tree-structured sequences with fixed utterances and branches restricts the scalability. To improve it, an architecture that generates a set of utterance patterns from a predefined set of topic categories (topic abstracted to some extent) is necessary in the future.

### 7.3 Desire in daily dialogue

In the developed system, the robot’s desire was incorporated into the dialogue system as a meta rule that regulates the behavior of the internal state that governs the behavior of the robot, and there was no explicit variable called desire. In other words, the desire (personal and social needs) was hard-coded in the system.

The BDI architecture that deals with a system desire can logically derive purposeful actions based on variable intentions and desires. But it is not suitable to derive each utterance from desires and intentions in daily dialogues since there is no clear purpose of a dialogue. The daily dialogue often follows typical patterns of dialogue flow, as in scripts, regardless of the speaker’s desire. The desire in the daily dialogue system should be designed as a driving force that globally guides the content of the dialogue although more specific desires that may occur at a certain moment.

### 7.4 Model of values

The internal state of the developed system includes the intentions, emotions, and memories, but the self-model and the external model (other person’s model) do not clearly appear in the architecture. A daily dialogue essentially involves the exchange of values among those who are talking. The values can be modeled by comparison between the robot’s self-model and the external model that describe what others are like and what the society is like. In the developed system, the robot’s preferences and values are hard-coded, and they do not arise from the robot’s experience. This not only causes less scalability of the system but also causes a person who is talking with the robot to not feel the reality of the preferences and values of the robot. For robots those engage in daily dialogues, an architecture that constructs a self-model along with the construction of an external model is essential.

### 7.5 Architecture for daily dialogue robot

As abovementioned, since a daily dialogue does not have a clear purpose, the robot needs to have the desire to initiatively continue the dialogue. In other words, unlike existing dialogue agents, it is necessary for daily dialogue that the robot should be an agent that is more independent of humans (not humans’ servant or tool). Here, we discuss the system architecture for that purpose.

**FIGURE 12:**
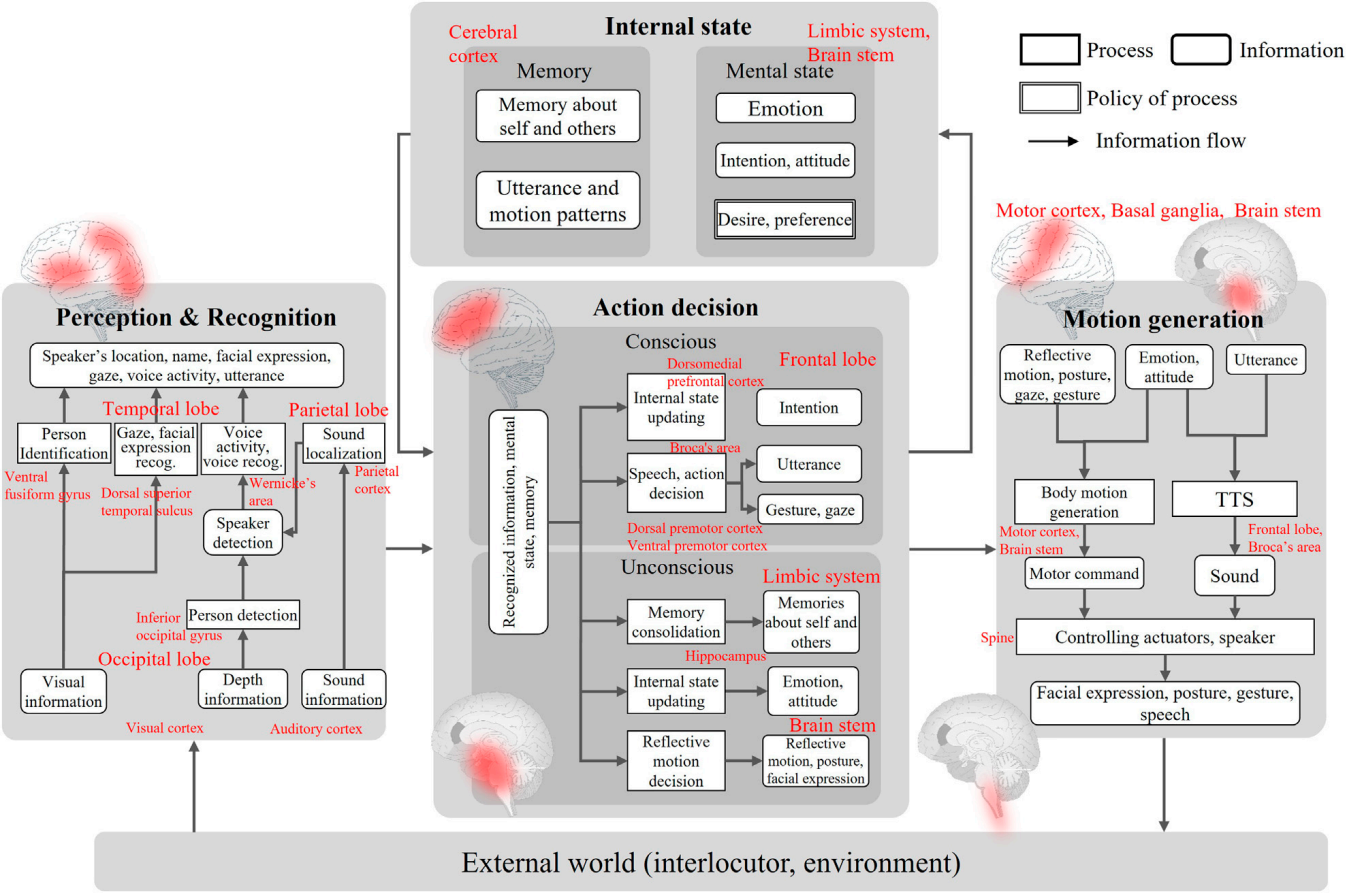
Architecture of the developed dialogue system.

Figure 12 summarizes the system architecture developed in this study. The architecture involves the perception/recognition part, action decision part, motion generation part, and internal state part, as well as the parts those most robot systems have (the perception/recognition part and motion generation part are not the focus of this study). Each process in the architecture roughly corresponds to a process in an area of the human brain. The system developed in a bottom–up manner has shown such correspondence. This means the developed dialogue robot system is valid as an autonomous system.

**FIGURE 13:**
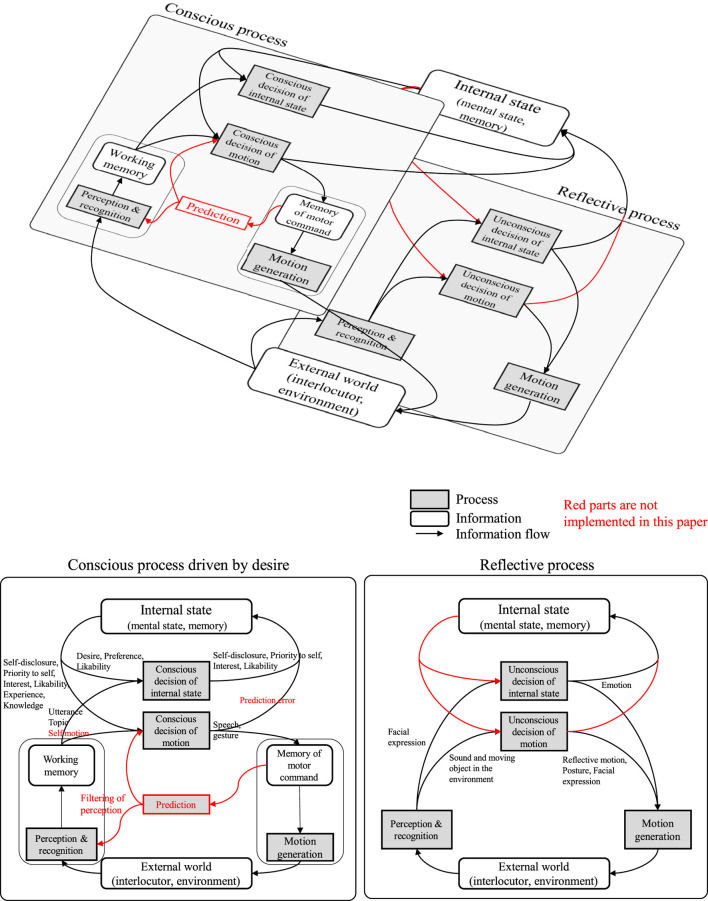
Dialogue system architecture in the viewpoint of conscious and reflective processes in action decision. Top is the hierarchical structure of two processes, and bottom is the detail of each process with the information flow.

In this architecture, the behavior generation and internal state updates are summarized into two processes: one is performed consciously (deliberatively) and the other is subconscious (reflexively). For example, the internal state of self-disclosure is consciously controlled, but the emotion is automatically updated. In addition, in the motion decision, the utterance is consciously controlled, but the facial actions are automatically expressed. Figure 13 redraws the system architecture in the viewpoint of conscious and reflective processes in the action decision. The process of consciously making a decision of the internal state has not been generally considered in the existing dialogue systems. It has been suggested that a daily dialogue system essentially needs decision-making of the internal states based on the system’s desires.

A hierarchical structure which consists of conscious and reflective behaviors has been considered in the control structure of conventional robots, but reflective behaviors have been less focused on in dialogue systems. This is because reflective behaviors during a dialogue were not assumed. In the developed system, there are conscious and subconscious processes, but there is no mediating function when the two processes conflict. Such a mediating system might be essential for a daily dialogue robot such that it has an agency and people feel its independence.

Another important factor for robot’s agency is the behavior based on the prediction of an agent’s observation and the prediction error. In conventional dialogue systems, a method for improving the recognition accuracy such that dialogue failure does not occur has been the focus, and the design of the interaction when a dialogue breakdown occurs has not been sufficiently considered. In the developed system, the topic was limited and the tree structure in the script was fixed in advance, so any unexpected behavior from the partner which causes a dialogue breakdown has to occur. Therefore, the system is designed to generate an interaction to recover from breakdown with natural behavior. In fact, our study has shown that such error recovery behavior can prevent people from losing their dialogue motivation (Uchida et al., 2019). To generalize, the system needs to have the capability to predict how the external world is changed by its behavior and compare it with the actual observation, which is not explicitly implemented in the developed system (shown in Figure 13 with red block and arrows). The prediction and prediction-error feedback corresponds to building the robot’s own model. In other words, the developed system lacks a structure for building a self-model. As abovementioned, the self and external world models are also required to form the values of the robot itself. Such an architecture needs to be studied such that the robot is recognized as a partner in daily dialogue in the future.

## 8 Limitations

As described in Section 1, daily dialogue involves mutual understanding and empathy (Okada et al., 1998) unlike task-oriented dialogue. The proposed system involves the capability to empathize with a dialogue partner, but the empathy that occurs in the dialogue is cognitive empathy rather than emotional empathy. This study assumes a first-meeting dialogue, and the dialogue maintained with only cognitive empathy in the experiment. But for long-term relationship building, the capability of emotional empathy is inevitable. It requires the estimation of psychological logic and emotion and adaptation to the other person, which should be implemented in the future system.

To realize humanlike, natural behavior of the robot, not only verbal interactions but also nonverbal interactions are important. In the proposed system, the android robot controls the eye gaze, facial expressions associated with emotions, interval at turn-taking, and gestures, but the model is simple compared to human behavior. The fact that the android robot could interact naturally even with nonverbal expressions in this system suggests that it could express at least a minimum level of humanlike behavior. But in MTT of this study, we asked the participants whether they comprehensively feel that a person is operating the robot or not. It could not analyze in detail whether humanlikeness was a linguistic or nonverbal factor, or conversely whether robot-likeness was a linguistic or nonverbal factor. It is necessary to evaluate the nonverbal humanlikeness, but it is difficult for the participant to answer questions such as “Do you think the robot’s gaze is controlled by a person?” In the future, it is necessary to develop an MTT evaluation method that can clarify the factors of humanness as well as develop rich nonverbal expressions of the android.

The MTT in this study does not compare the autonomous android with the tele-operated android. Therefore, the evaluation is based on the participants’ beliefs about the autonomous robot’s performance. In the Turing test, the evaluations were made by comparing two conversations, one with an AI and one with a human, and it has been revealed that evaluators can find the difference between humans and AIs based on the slight differences of conversation flow (Jacquet et al., 2019). This has suggested that it is difficult to judge whether the android robot is autonomously controlled or tele-operated. In other words, by comparing autonomous and tele-operated androids, it is possible to investigate what factors make humanlikeness and what factors bring unnaturalness. Because of the hardware difficulties in making an android robot operate in the same way as a human, this study has adopted an evaluation method without comparison. However, in terms of clarifying the factors of humanlike characteristics, comparative evaluation of autonomous and tele-operated systems is necessary in the future study.

## 9 Summary

In task-oriented dialogues, information that the system should acquire and provide to the user are clearly defined in order to achieve the desired task. This makes it possible to determine the quality of the system’s utterances and generate the necessary utterances based on task evaluation. On the other hand, in daily dialogues, it is not possible to evaluate whether or not the goal is clearly approached by each utterance because the necessary information is not clearly defined. In this study, in comparison to human daily dialogue, we considered that it is necessary to generate utterances based on the robot’s desires and internal state in daily dialogue. Then, instead of controlling the utterance content for each turn of dialogues, we implemented a method to control the content of the topic based on the robot’s internal state. The developed system could continue daily dialogues for about 10 min in the scene where the robot and partner met for the first time in the experiment. This result suggests that the system construction method assumed in this study is an effective approach to realize daily dialogue. The suggestions for the development of the daily dialogue robot are summarized as follows:

1) Dialogue control based on the system’s own desires and intentionally controlled internal states are necessary.
2) A relationship between the dialogue partners should be the focus of daily dialogues rather than conveyed information through dialogue.
3) By controlling the topic described by the script in which the utterance is designed in advance, it is possible to continue the daily dialogue while suppressing the dialogue failure.
4) In order to improve the scalability of the system, it is desirable to develop a method to generate the scripts from topic content.

In this study, the recognition and nonverbal expression system were not mainly focused on, but they are also important in the daily dialogue system. It is important to recognize not only the voice, emotions, and attitudes of the dialogue partner but also the preferences, opinions, and the intention of the partner. Uchida et al. (2021) revealed that a dialogue strategy that estimates the preferences of the dialogue partner enhances the partner’s motivation for dialogue. By incorporating such a system, a more natural daily dialogue can be realized. In addition, regarding nonverbal expressions, not only facial expressions and gestures but also gaze, filler, speech level, and timing of taking speaking turns are controlled that reflect the intention of the system. Although they are implemented in this system, it is possible to realize more natural daily dialogue by further improving these nonverbal expressions.

## Data Availability

The raw data analyzed in this article is available from the corresponding author on reasonable request.
