# Supplementary material for: A study of interactive robot architecture through the practical implementation of conversational android
Source: Front Robot AI. 2022 Oct 11;9:905030. doi: 10.3389/frobt.2022.905030 (PMC9592984; doi:10.3389/frobt.2022.905030)
Supplement: Supplementary file 1 [file DataSheet1.docx]

Supplementary Material


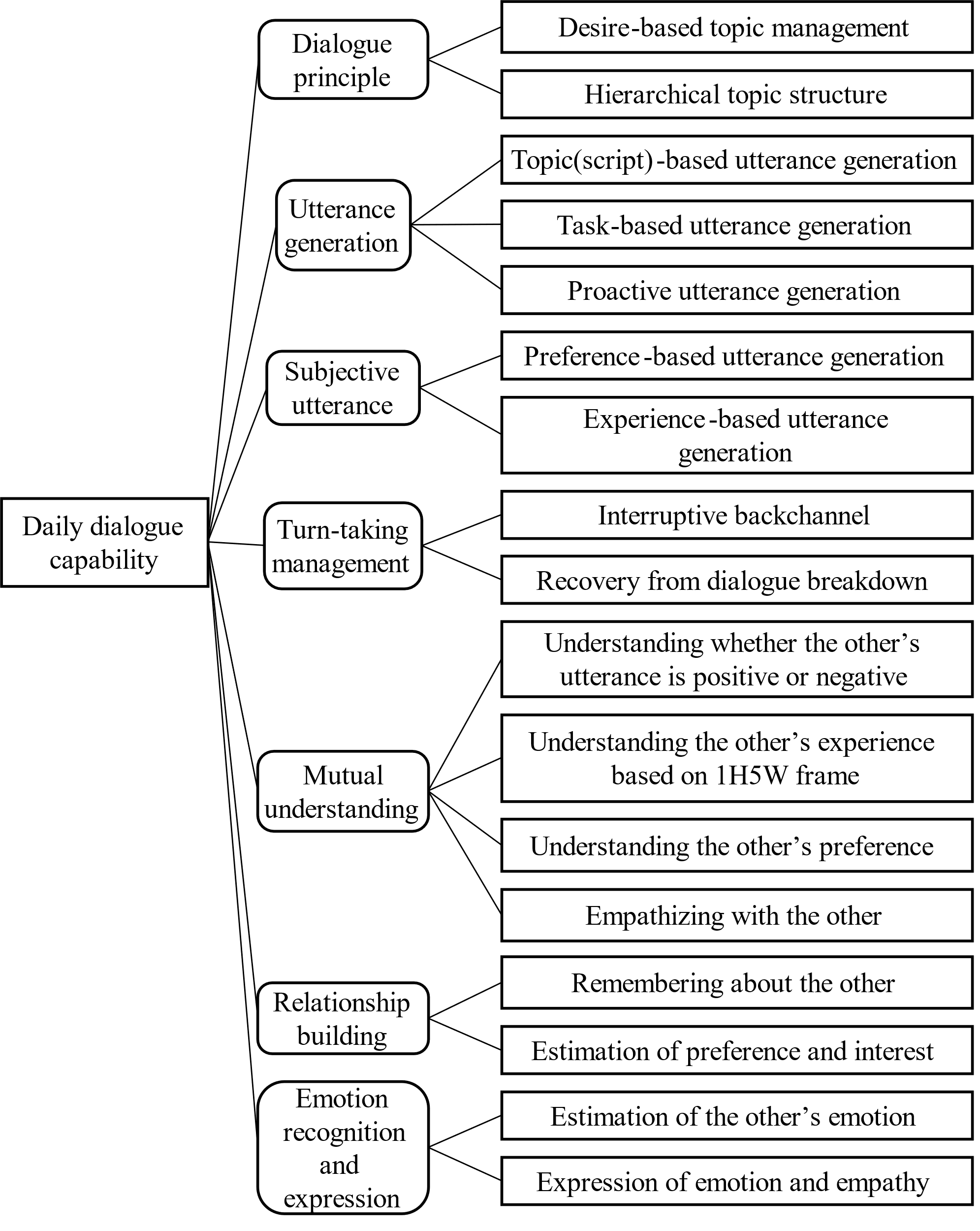


**Supplementary Figure 1.** The functions needed for daily dialogue. This summarizes the functions needed for daily dialogue described in the manuscript, which have been less focused on in task-oriented dialogue systems. Different from task-oriented dialogue system, a relationship between dialogue partners should be focused on in daily dialogue rather than merely conveying information.


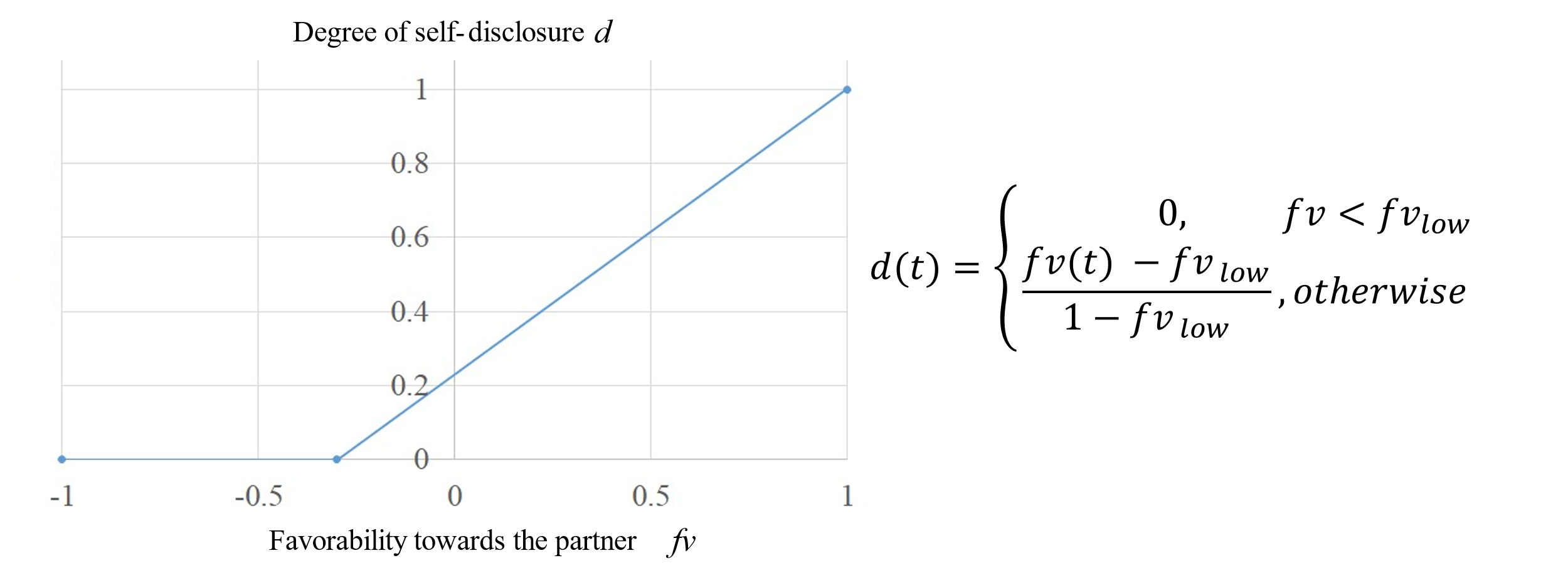


**Supplementary Figure 2.** The robot’s self-disclosure determined based on the favorability towards the partner. *d* is the degree of self-disclosure and *f_v_* is the favorability of the robot towards the partner. The degree of self-disclosure is basically proportional to the favorability but becomes minimum when the favorability is too much low.


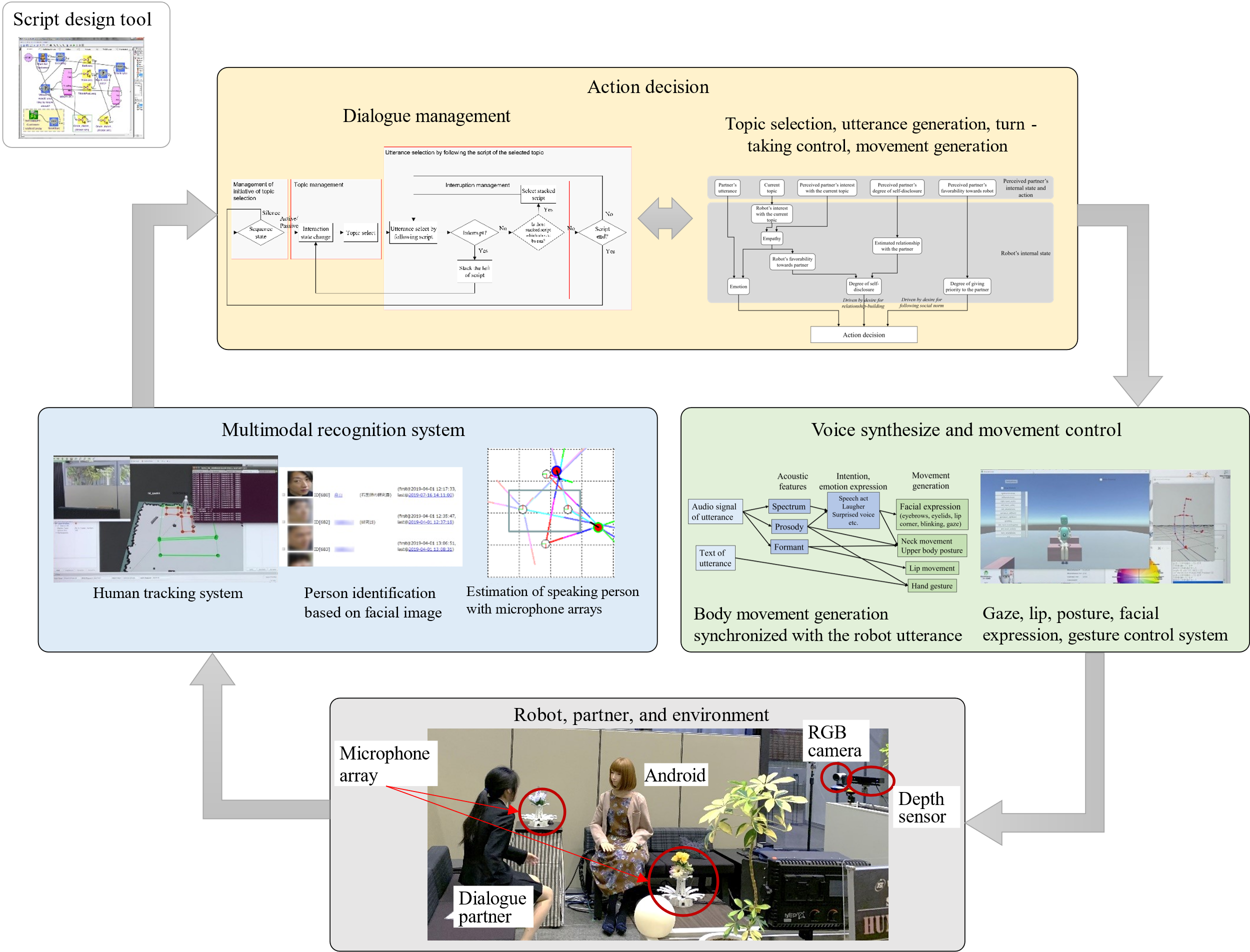


**Supplementary Figure 3.** A total system to control the dialogue android robot


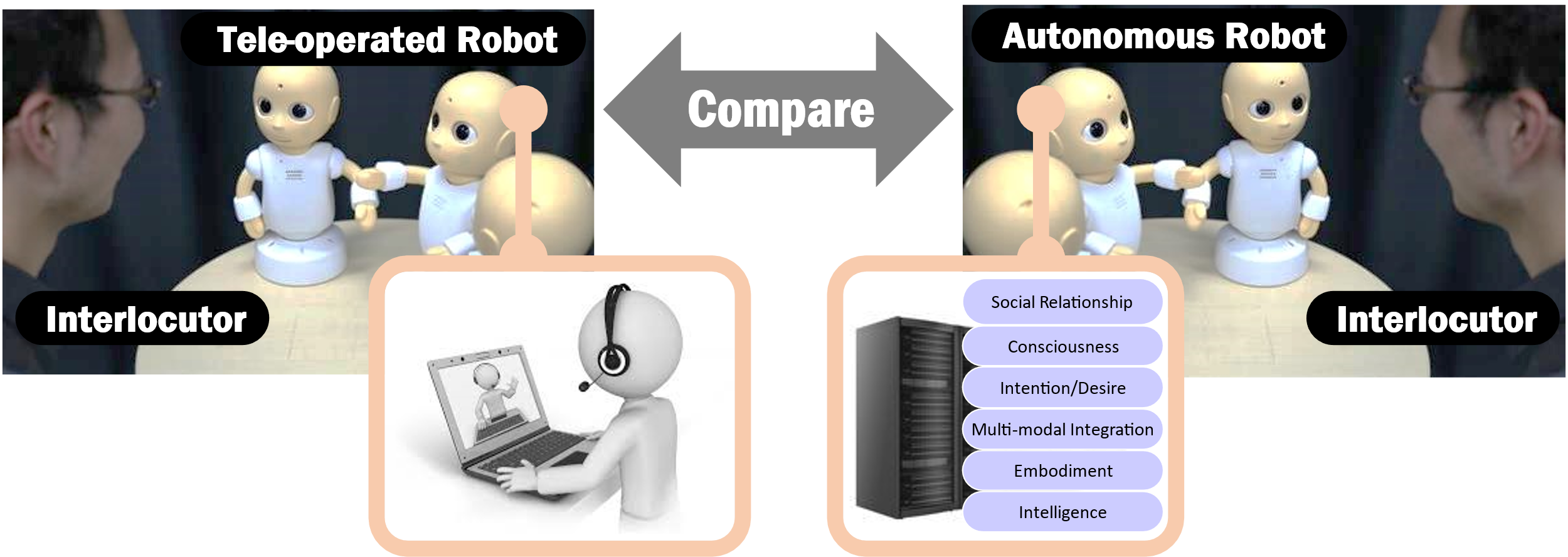


**Supplementary Figure 4.** A concept of Multimodal Turing Test

**Supplementary Table 1.** Existing dialogue systems summarized with respect to the functions needed for daily dialogue. A: Yimr [Thorisson 1999], B: SimSensei [DeVault et al., 2014], C: Mac model [Ushida et al., 1998], D: Counseling for cyberbullying [Zwaan et al., 2012], E: CALO [Yorke-Smith et al., 2012], F: Chat system with context understanding [Narimatsu et al., 2019], G: DAC-h3 [Moulin-Frier et al., 2018], H: Eva [Kasap and Magnenat-Thalmann 2011], I: Dialogue with preference and relations [Kobayashi and Hagiwara 2016], J: Kismet [Breazeal 2003], K: Blender [Smith et al., 2020], L: Max [Kopp et al., 2005], M: Our system

|  |  | A | B | C | D | E | F | G | H | I | J | K | L | M |
| --- | --- | --- | --- | --- | --- | --- | --- | --- | --- | --- | --- | --- | --- | --- |
| Dialogue principle | Desire-based topic management |  |  | ✓ | ✓ | ✓ |  | ✓ |  |  | ✓ |  | ✓ | ✓ |
|  | Hierarchical topic structure |  |  |  |  |  |  |  |  |  |  |  |  | ✓ |
| Utterance generation | Topic(script)-based utterance generation |  |  |  |  |  |  |  |  |  |  |  |  | ✓ |
|  | Task-based utterance generation | ✓ | ✓ | ✓ | ✓ | ✓ |  |  | ✓ |  |  |  | ✓ | ✓ |
|  | Proactive utterance generation | ✓ |  | ✓ |  | ✓ |  | ✓ | ✓ |  | ✓ |  | ✓ | ✓ |
| Subjective utterance | Preference-based utterance generation |  |  |  |  |  |  |  |  |  |  | ✓ |  | ✓ |
|  | Experience-based utterance generation |  |  |  |  |  |  |  | ✓ |  |  | ✓ | ✓ | ✓ |
| Turn-taking management | Interruptive backchannel | ✓ |  |  |  |  |  |  |  |  |  |  |  | ✓ |
|  | Recovery from dialogue breakdown | ✓ |  | ✓ |  |  |  |  |  |  |  |  |  | ✓ |
| Mutual understanding | Understanding polarity of the other’s utterance |  | ✓ |  | ✓ |  | ✓ |  |  | ✓ |  | ✓ | ✓ | ✓ |
|  | Understanding the other’s experience based on 1H5W frame |  |  |  |  |  | ✓ |  |  | ✓ |  |  |  | ✓ |
|  | Understanding the other’s preference |  |  |  |  |  |  |  |  | ✓ |  |  | ✓ | ✓ |
|  | Empathizing with the other |  | ✓ |  |  |  | ✓ |  |  |  |  |  | ✓ | ✓ |
| Relationship Building | Remembering about the other |  |  | ✓ | ✓ | ✓ |  |  | ✓ | ✓ |  |  | ✓ | ✓ |
|  | Estimation of preference and interest |  |  |  |  |  |  |  |  | ✓ |  |  |  | ✓ |
| Emotion recognition and expression | Estimation of the other’s emotion |  | ✓ |  |  |  |  |  |  |  |  |  | ✓ | ✓ |
|  | Expression of emotion and empathy | ✓ | ✓ | ✓ | ✓ |  | ✓ |  | ✓ | ✓ | ✓ |  | ✓ | ✓ |

**Supplementary Table 2.** Examples of topic for each sequence state and interaction state

|  | | **Sequence state** | | | | |
| --- | --- | --- | --- | --- | --- | --- |
|  |  | Idle | InitialTalk | Dialogue | Exception | Close |
| **Interaction state** | Active | Monologue | Asking partner’s job | Asking partner’s hobby | None | None |
|  | Passive | Starting talk  (ex. calling robot’s name) | Answering a question about name | Answering a question about hobby | Unable to answer a question /  no selectable topic | Partner has left |
|  | Silence | None | None | None | None | None |

**Supplementary Table 3.** The robot’s speech style owing to the social relationship between the robot and partner

|  | | **Social status of partner** | | |
| --- | --- | --- | --- | --- |
|  |  | Subordinate  (ex. child) | Close  (ex. same generation, colleague) | Superior  (ex. boss, older generation) |
| **Degree of relationship** | High  (>=0.8) | Not assumed | Level 3 | Level 2 |
|  | Middle  (<0.8 & >=0.05) | Not assumed | Level 2 | Level 1 |
|  | Low | Not assumed | Leve 1 | Level 1 |
